# Supplementary material for: Dual-color DNA-PAINT single-particle tracking enables extended studies of membrane protein interactions
Source: Nat Commun. 2023 Jul 19;14:4345. doi: 10.1038/s41467-023-40065-8 (PMC10356854; doi:10.1038/s41467-023-40065-8)
Supplement: Supplementary file 1 — Supplementary Information [file 41467_2023_40065_MOESM1_ESM.pdf]

Supplementary Information

Dual-color DNA-PAINT single-particle tracking enables extended studies of membrane protein interactions

Supplementary Note 1. DNA sequences

DNA-sequences are in 5'-3' notation.

- DNA-PAINT-SPT sequences

  Imager strand A, Cy3B-conjugated: 4x(GA) - Cy3B
  GAGAGAGA - Cy3B

  Imager strand B, ATTO643-conjugated: 4x(GT) - ATTO643
  GTGTGTGT - ATTO643

  Docking strand A: BG- or azide- conjugated: 37.5x(TC)
  BG/azide - TCTCTCTCTCTCTCTCTCTCTCTCTCTCTCTCTCTCTCTCTCTCTCTCTCTCTCTCTCTCTCTCTCTCTCTCTCT

  Docking strand B: BG- or azide-conjugated: 37.5x(AC)
  BG/azide - ACACACACACACACACACACACACACACACACACACACACACACACACACACACACACACACACACACACACACA

- Single-dye DNA sequences
  We used a single-dye control designed to be of similar size and mass compared to a 50% imager strand-occupied DNA-PAINT-SPT docking strand. The single-dye docking-imager strand pair consists of a 75 nucleotide docking strand and a 35 nucleotide fully complementary imager strand. At our experimental conditions (ionic strength, temperature, timescale), the imager strand is considered to be irreversibly bound to the docking strand.

  Imager strand A:
  ATAATAAGTAATCTACAACAATCGGGTGGGTCAGC - Cy3B

  Imager strand B:
  TAATGAAATGGGAACTAACTCTCGGAAACCTTTAT - ATTO643

  Docking strand A:
  BG - TTTTTTTTTTATAAAGGTTTCCGAGAGTTAGTTCCCATTTCATTATTTTTTTTTTTTTTTTTTTTTTTTTTTTTT

  Docking strand B:
  BG - TTTTTTTTTTGCTGACCCACCCGATTGTTGTAGATTACTTATTATTTTTTTTTTTTTTTTTTTTTTTTTTTTTTT

Supplementary Note 2. Analytical derivation of dimer concentration for ligand-induced homodimerization

We briefly outline the analytical derivation of the dimer concentration for ligand-induced homodimerization. For a detailed discussion refer to Binder et al., 2021 [1]. We use 2D densities in 1 μm^{-2} and convert molar 3D concentrations into 1 μm^{-3}, where

$$1\,\mu\text{M} = \frac{1 \times 10^{-6} \cdot 6.022 \times 10^{23}}{1\,\text{L}} = \frac{6.022 \times 10^{17}}{1 \times 10^{15}\,\mu\text{m}^3} = 602.21\,\mu\text{m}^{-3}$$

Consider a system with ligands in solution with concentration c_L and protein monomers embedded in a lipid bilayer with surface concentration Γ_M. In this system, the ligands can bind up to two monomers simultaneously. We consider both the equilibrium of a monomer binding to a free ligand eq. (Eq. 1) and the equilibrium of a monomer binding to an already bound ligand eq. (Eq. 2):

$$\mathrm{M} + \mathrm{L} \rightleftharpoons \mathrm{ML}, \qquad \textbf{(Eq. 1)}$$

and

$$\mathrm{ML} + \mathrm{M} \rightleftharpoons \mathrm{MLM}. \qquad \textbf{(Eq. 2)}$$

The dissociation constant K_B for a monomer bound to a single ligand, ML, is defined as

$$K_B = \frac{\Gamma_M c_L}{\Gamma_{ML}}, \qquad \textbf{(Eq. 3)}$$

where Γ_{ML} is the surface concentration of ML. The dissociation constant K_X for the ligand-homodimer complex, MLM, is defined as

$$K_X = \frac{\Gamma_{ML}\Gamma_M}{\Gamma_{MLM}}, \qquad \textbf{(Eq. 4)}$$

where Γ_{MLM} is the concentration of MLM.
To derive an expression for Γ_{MLM} that does not include Γ_M or Γ_{ML}, we need the equation for the mass balance of the protein monomer,

$$\Gamma_{M,\mathrm{tot}} = \Gamma_M + \Gamma_{ML} + 2\Gamma_{MLM}. \qquad \textbf{(Eq. 5)}$$

Combining eqs. (Eq. 3) to (Eq. 5) and rearranging, we obtain

$$\Gamma_{MLM} = \frac{K_X(K_B+c_L)^2 + 4K_B\Gamma_{M,\mathrm{tot}}c_L - \sqrt{K_X}(K_B+c_L)\sqrt{K_X(K_B+c_L)^2 + 8K_B\Gamma_{M,\mathrm{tot}}c_L}}{8K_Bc_L}, \qquad \textbf{(Eq. 6)}$$

where we have discarded the second solution in which the second term (containing the square root) is positive, as it leads to negative values of Γ_M and Γ_{ML}.
The density of reconstituted proteins $\Gamma_M = \Gamma_{\text{M, channel 1}} + \Gamma_{\text{M, channel 2}}$ is measured by counting receptors before adding the dimerization ligand, since the random association of magenta and green labeled receptors into same-color and dual-color dimers (total: 25% magenta-magenta, 25% green-green, 50% magenta-green) would otherwise change the apparent receptor density. One hour after adding ligands, the number of dimers is determined by detecting co-diffusion trajectories of dual-color dimers. The number of apparent dimers is multiplied by the quotient of the lower density over the total density of green and magenta receptors: $\Gamma_M \cdot \min(\Gamma_{\text{M, channel 1}}, \Gamma_{\text{M, channel 2}})^{-1} \approx 2$. The fraction of dimerized molecules is then fit by eq. (Eq. 6) with free parameters K_X, K_B and a correction factor accounting for unlabeled molecules.

Supplementary Note 3. Surface passivation

The high concentration of fluorescently labeled imager strands in solution poses two challenges: imager strands bind to the surface via unspecific interactions with the surface or cell debris, and they contribute to a diffuse background, decreasing the signal-to-background ratio. We screened various surface passivation methods (see Supplementary Fig. 11, Supplementary Table 1) and found lipid bilayers to provide the best passivation efficiency. Cell attachment is ensured by functionalizing the bilayers with cell adhesion promoting molecules (RGD, PEG-RGD functionalized lipids or His-tagged ICAM-1 reconstituted on nickelated lipids). Bilayers containing PEG-RGD functionalized lipids or RGD-functionalized lipids had comparable passivation efficiencies, but PEG-RGD functionalized lipids performed better in terms of cell attachment. We also used PLL-PEG-RGD successfully for single-color experiments with Cy3B. When used with ATTO643-labeled imager strands, significant binding of imager strands to the surface was observed, which could at least partially be prevented by adding Trolox/Trolox-quinone as a reductant and oxidant system[2] (see Supplementary Fig. 1). During the live cell imaging time scales typical for SPT experiments (minutes to max. two hours), we observed no significant effects on cell viability or morphology beyond the differences in cell adhesion to the surfaces.

Surface	Imager strand	Localizations (background) [µm^{-2}]	Localizations (cells) [µm^{-2}]	Ratio
BSA	ATTO643	0.67	0.78	1.16
	Cy3B	0.25	0.37	1.44
Fibronectin	ATTO643	0.30	0.35	1.17
	Cy3B	0.17	0.22	1.33
PLL	ATTO643	0.17	0.30	1.81
	Cy3B	0.04	0.15	4.07
PLL-PEG-RGD	ATTO643	0.05	0.34	6.48
	Cy3B	0.06	0.37	6.45
DSPE-RGD	ATTO643	0.01	0.12	9.21
	Cy3B	0.01	0.08	11.08
ICAM	ATTO643	0.01	0.11	15.04
	Cy3B	0.01	0.10	12.34
DSPE-PEG-RGD	ATTO643	0.01	0.15	13.05
	Cy3B	0.01	0.11	16.53

Supplementary Table 1. Localizations on background and cells for different surface passivation methods

Supplementary Figures

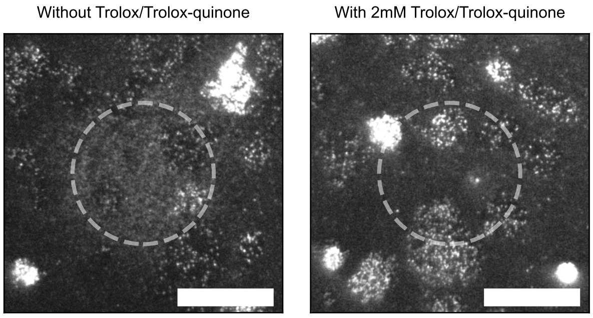

Supplementary Figure 1. ATTO643 photoreaction on PLL-PEG-RGD with and without Trolox.
Cells labeled with DNA-PAINT and 40 nM ATTO643-labeled imager strands. First frame after illuminating for 1 min in the center circular region, with 2 mM Trolox/Trolox-quinone (left), or without Trolox/Trolox-quinone in the imaging buffer (right). This experiment was repeated independently two times with similar results. Scale bar: 10 µm.

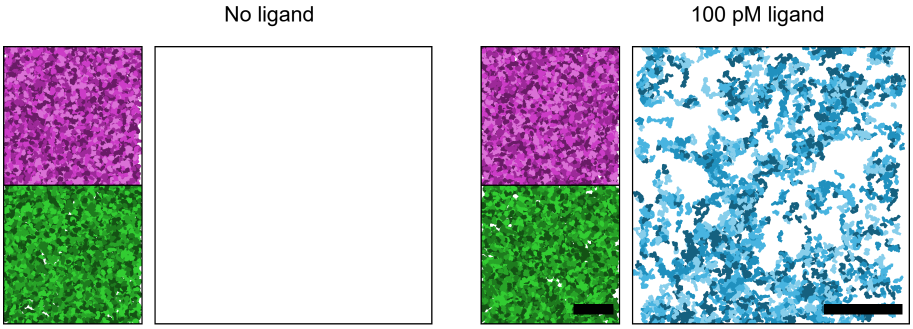

Supplementary Figure 2. Absence of interaction or crosslinking artifacts introduced by DNA-PAINT-SPT labels.
DNA-PAINT-SPT single-molecule trajectories collected during 40 second recordings of reconstituted FKBP proteins with and without dimerization agent AP20187. Small panels (magenta and green) are single-molecule trajectories collected in two color channels and big panel (blue) are detected co-diffusion events that exceed 10 frames (400 ms). The absence of detectable dimers when no ligand is present, suggests that the DNA-PAINT-SPT label itself does not introduce interactions or crosslinking artifacts. This experiment was repeated independently three times with similar results. Scale bars: 20 µm.

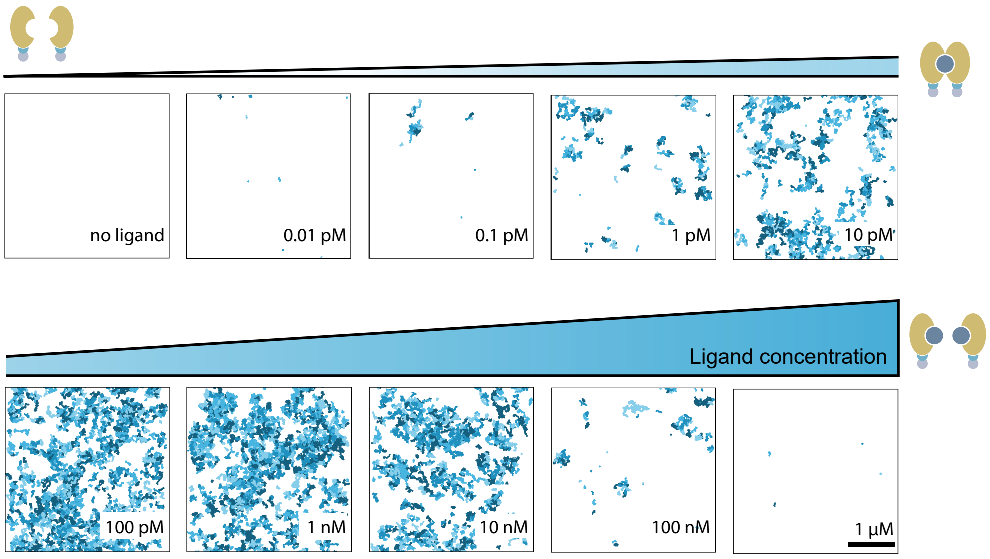

Supplementary Figure 3. Dimerization of FKBP proteins during ligand-titration experiments from onset to suppression of interaction.
Co-diffusion events exceeding 10 frames (400 ms) at increasing concentrations of dimerization agent AP20187, detected by dual-color DNA-PAINT-SPT of reconstituted FKBP proteins. At concentrations above 10 nM, monomers are saturated with ligands and interactions are suppressed. This experiment was repeated independently three times with similar results. Measurement durations are 40 s. Scale bar: 20 µm.

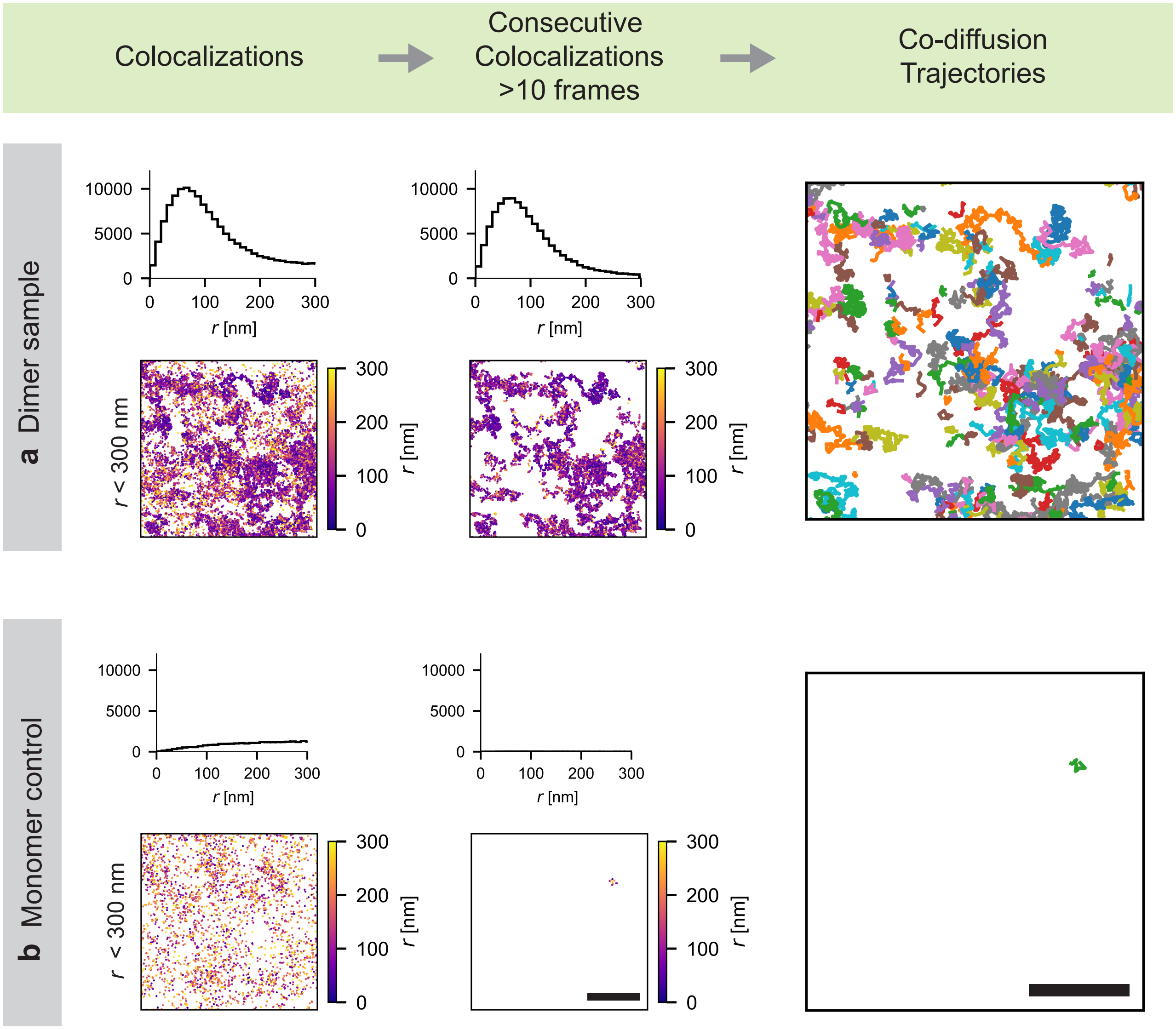

Supplementary Figure 4. Interaction analysis framework.
Detected colocalizations in a sample of reconstituted FKBP dimers (**a**), top, 100 pM dimerizing agent) and FKBP monomer control (**b**), bottom, no dimerizing agent) during a 20 s measurement. Left column shows colocalizations, color-coded by the distance r of each individual colocalizing pair, and a histogram of their pairwise distances. A colocalization threshold of 300 nm was used. Middle column shows remaining consecutive colocalizations and the respective pairwise distance histogram, after tracking and discarding all trajectories shorter than 10 frames (400 ms). Right column shows co-diffusion trajectories with gaps of up to 6 frames closed, color-coded by their trajectory ID. Scale bar is 10 µm. Source data are provided.

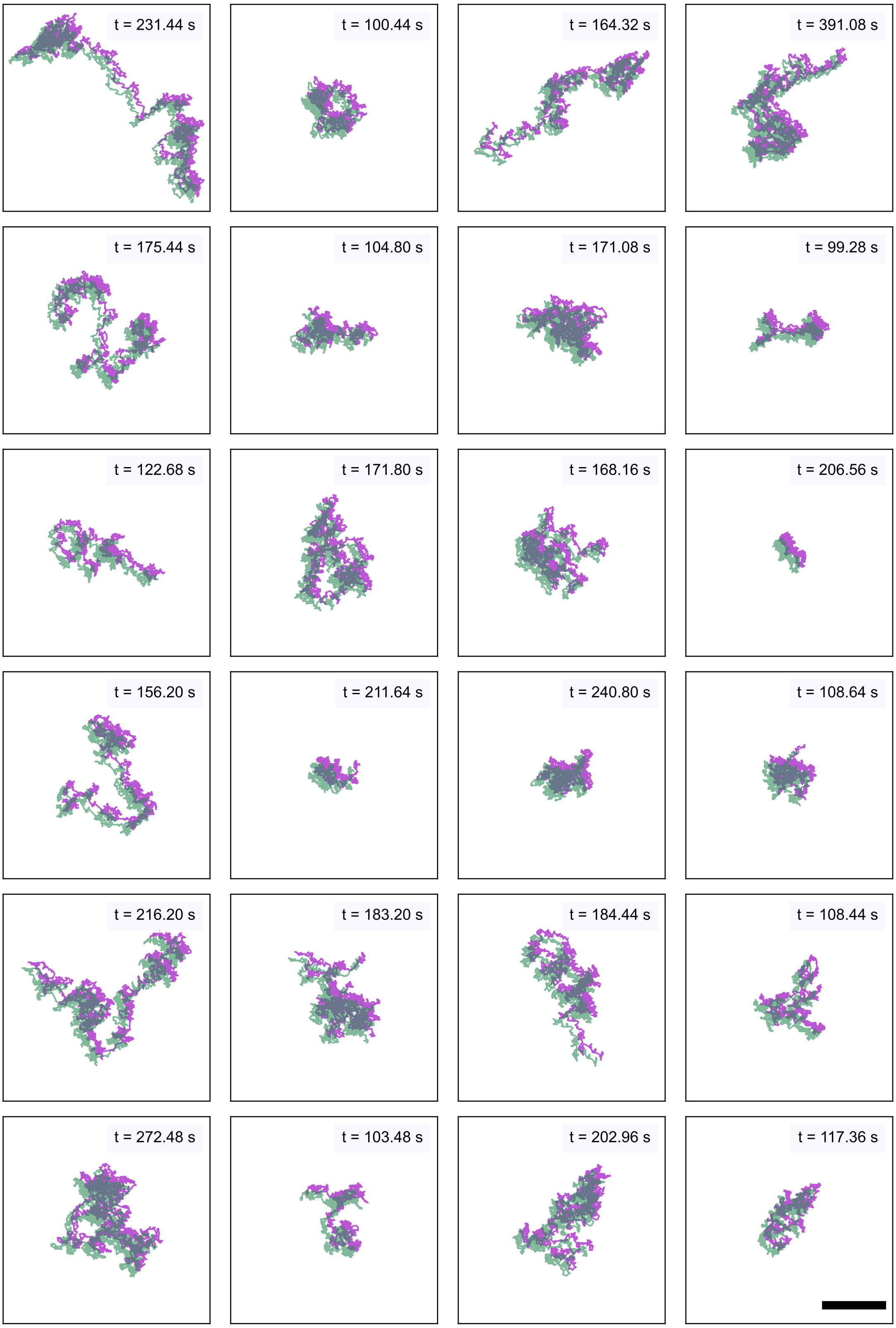

Supplementary Figure 5. Ligand-induced FKBP dimers detected with dual-color DNA-PAINT-SPT.
The longest trajectories (30th-percentile) of co-diffusing AP20187-induced FKBP dimers, labeled with dual-color DNA-PAINT and recorded during a 15 minute TIRFM measurement. For displaying purposes, tracks were moved in opposite x and y directions by 1 µm. Scale bar: 10 µm.

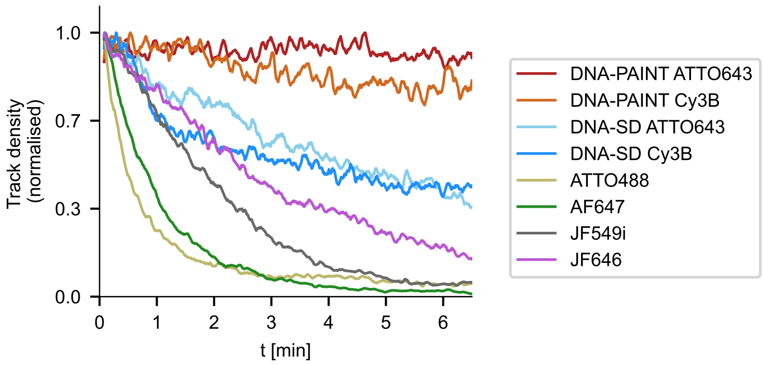

Supplementary Figure 6. Photostability of different labeling methods and fluorophores in in vitro experiments.
Number of trajectories per frame of reconstituted FKBP proteins labeled with DNA-PAINT (ATTO643-imager strands in red, $n_{\text{samples}} = 3$; Cy3B-imager strands in orange, $n_{\text{samples}} = 3$), single-dye DNA (ATTO643-fluorophore in light blue, $n_{\text{samples}} = 4$; Cy3B-fluorophore in dark blue, $n_{\text{samples}} = 4$) or single BG-conjugated fluorophores (ATTO488 in yellow, $n_{\text{samples}} = 2$; AlexaFluor647 in green, $n_{\text{samples}} = 2$; JF549i in gray, $n_{\text{samples}} = 5$; JF646 in magenta, $n_{\text{samples}} = 5$). Source data are provided.

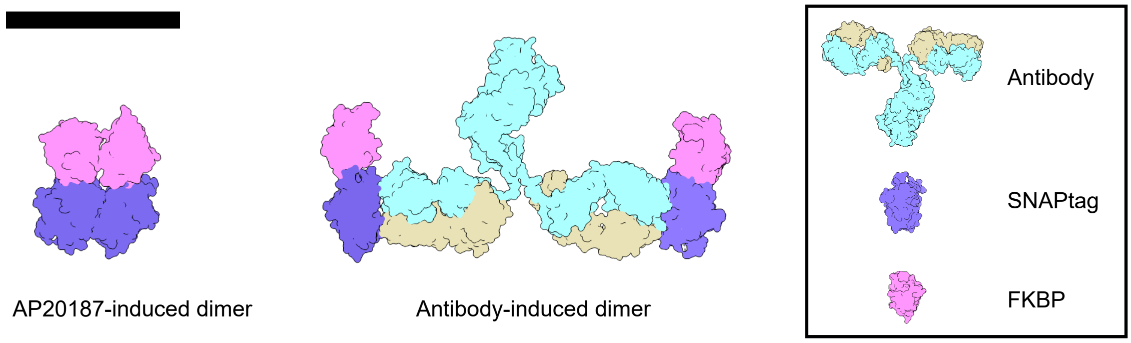

Supplementary Figure 7. Dimerization geometry for ligand- and antibody-induced dimerization.
Size comparison of FKBP-SNAPtag complexes dimerized via the ligand AP20187 and an anti-SNAPtag antibody. Protein structures from PDB (SNAPtag: 3KZY, FKBP$^{\text{f36v}}$: 1BL4, Antibody: 1IGT). Scale bar: 10 nm.

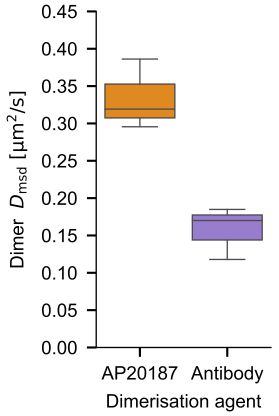

Supplementary Figure 8. Diffusion constants of dimers for AP20187- and antibody-induced dimerization.
Diffusion constants derived from mean-square displacement of co-diffusing trajectories show a two-fold reduction for antibody-induced dimers ($D_{\text{msd, AP20187}} = 0.33 \pm 0.05\,\mu\text{m}^2$/s, $D_{\text{msd, Antibody}} = 0.16 \pm 0.04\,\mu\text{m}^2$/s). This effect is likely due to the bigger size and mass of the antibody-induced dimer, and potentially also by the increased coupling of thermal energy to the rotational degree of freedom, as the antibody-induced dimer has a higher moment of rotational inertia compared to the compact ligand-induced dimer. Boxes, line and whiskers show, respectively, 25–75 quartiles, median, and minimum and maximum values of diffusion constants. Data from three samples for each condition. Source data are provided.

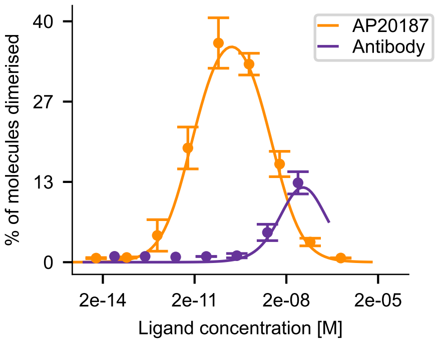

Supplementary Figure 9. Dimerization curve of ligand-titration experiments measured using DNA-PAINT-SPT. Fraction of dimerized molecules as detected using DNA-PAINT-SPT during ligand-titration experiments. AP20187 or anti-SNAPtag antibody were used to induce dimerization. Labeled fractions according to the fit were $60 \pm 17\,\%$. Error bars denote mean $\pm$ standard deviation of data collected from three field of views of each sample of a titration series for each dimerization agent. Source data are provided.

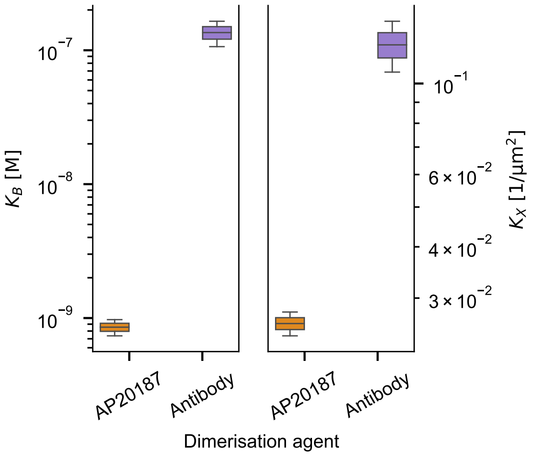

Supplementary Figure 10. 2D dissociation constants of different dimerization agents measured using DNA-PAINT-SPT. Dissociation constants K_X and K_B of AP20187 and anti-SNAPtag antibody interaction with FKBP^{f36v} as determined from fitting the fraction of dimerized molecules. Labeled fractions according to the fits were $67 \pm 16\,\%$. Boxes, line and whiskers show, respectively, 25–75 quartiles, median, and minimum and maximum values of dissociation constants. The data used for fitting was collected from three field of views of each sample of a titration series, prepared in duplicates for each dimerization agent. Source data are provided.

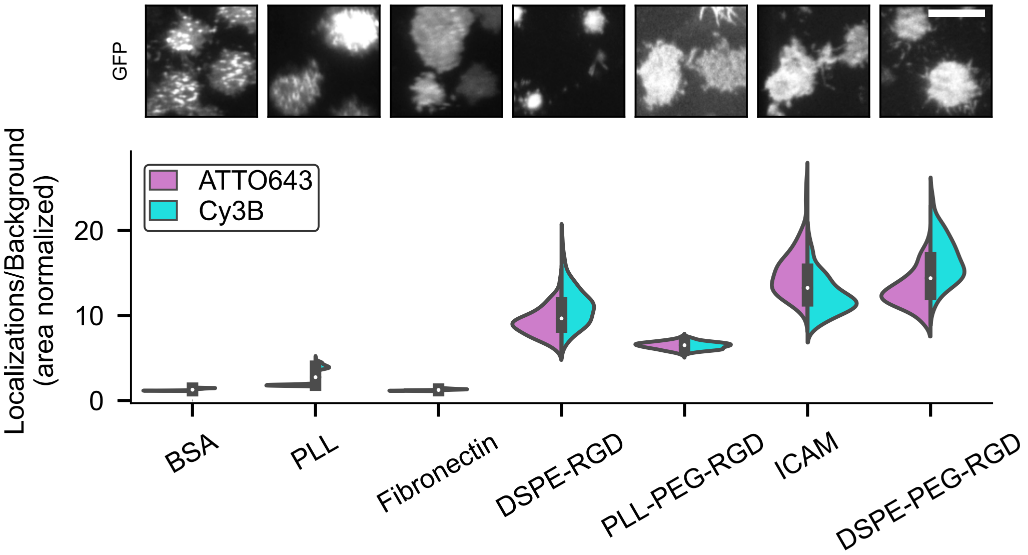

Supplementary Figure 11. Screening of passivation methods.
Ratio of localizations detected on DNA-PAINT labeled cells with 40 nM imager strands (Cy3B- or ATTO643-conjugated) per area versus localizations detected outside of cells per area, for different surfaces. Upper row shows GFP signal of adhered cells (scale bar: 10 µm). See Supplementary Movie 4 for video version with single-molecule channels. Boxes, line and whiskers overlayed over the violin plot show, respectively, 25–75 quartiles, median, and minimum and maximum values. Data and panels from one representative field of view for each condition. Source data are provided.

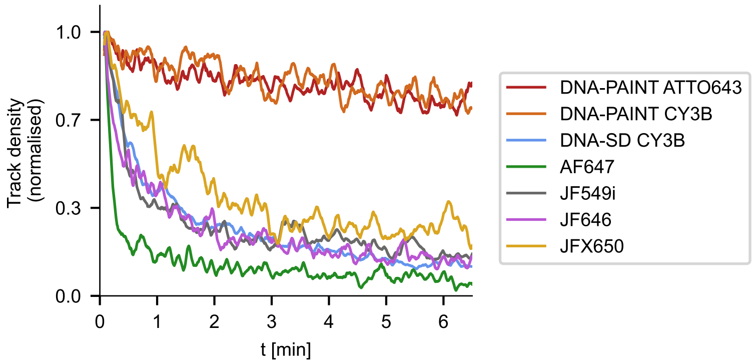

Supplementary Figure 12. Trajectory density over time for DNA-PAINT labels and single-dye controls.
Number of trajectories per frame on individual cells labeled with DNA-PAINT (ATTO643-imager strands in red, $n_{\text{cells}} = 25$; Cy3B-imager strands in orange, $n_{\text{cells}} = 25$), single-dye DNA (Cy3B-fluorophore in blue, $n_{\text{cells}} = 32$) or single BG-conjugated fluorophores (AlexaFluor647 in green, $n_{\text{cells}} = 7$; JF549i in gray, $n_{\text{cells}} = 15$; JF646 in magenta, $n_{\text{cells}} = 6$; JFX650 in yellow, $n_{\text{cells}} = 6$). Source data are provided.

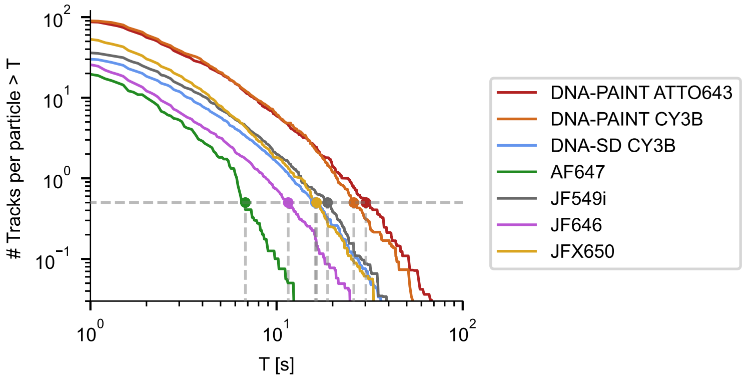

Supplementary Figure 13. Tracks per particle over time for DNA-PAINT labels and single-dye controls. Single-molecule trajectories with a duration longer than T, normalised to the initial number of trajectories per individual cell. Membrane proteins are labeled with DNA-PAINT (ATTO643-imager strands in red, $n_{\text{cells}} = 25$; Cy3B-imager strands in orange, $n_{\text{cells}} = 25$), single-dye DNA (Cy3B-fluorophore in blue, $n_{\text{cells}} = 32$) or single BG-conjugated fluorophores (AlexaFluor647 in green, $n_{\text{cells}} = 7$; JF549i in gray, $n_{\text{cells}} = 15$; JF646 in magenta, $n_{\text{cells}} = 6$; JFX650 in yellow, $n_{\text{cells}} = 6$). Source data are provided.

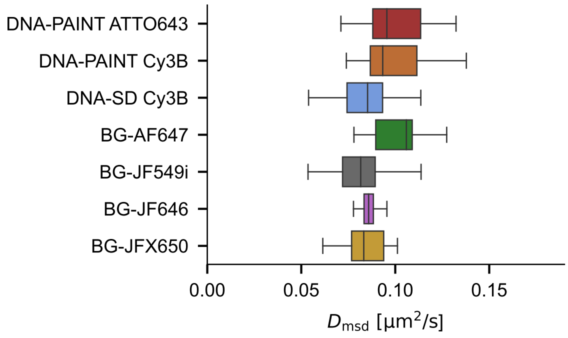

Supplementary Figure 14. Diffusion constants of membrane proteins labeled with DNA-PAINT and single dye probes. Mean-square displacement derived diffusion constants of membrane proteins on cells labeled with DNA-PAINT (ATTO643-imager strands in red, $n_{\text{cells}} = 25$; Cy3B-imager strands in orange, $n_{\text{cells}} = 25$), single-dye DNA (Cy3B-fluorophore in blue, $n_{\text{cells}} = 32$) or single BG-conjugated fluorophores (AlexaFluor647 in green, $n_{\text{cells}} = 7$; JF549i in gray, $n_{\text{cells}} = 15$; JF646 in magenta, $n_{\text{cells}} = 6$; JFX650 in yellow, $n_{\text{cells}} = 6$). Boxes, line and whiskers show, respectively, 25–75 quartiles, median, and minimum and maximum values of diffusion constants. Source data are provided.

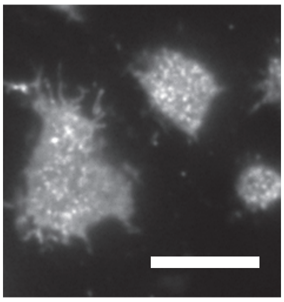

Supplementary Figure 15. Accessibility of docking strands. Average fluorescence intensity during a 80 s measurement of Jurkat T-cells with densely labeled membrane proteins using DNA-PAINT docking strands and Cy3B-conjugated imager strands, to visualise potential exclusion effects of DNA-labeled proteins from cell-surface contacts. This experiment was repeated independently five times with similar results.

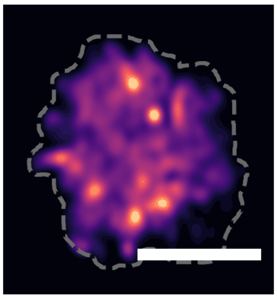

Supplementary Figure 16. Trajectory density variation across cell surface.
Density of single-molecule trajectories across Jurkat T-cell surface during a 400 s measurement for DNA-PAINT docking strand labeled membrane proteins and Cy3B-conjugated imager strands. This experiment was repeated independently five times with similar results.

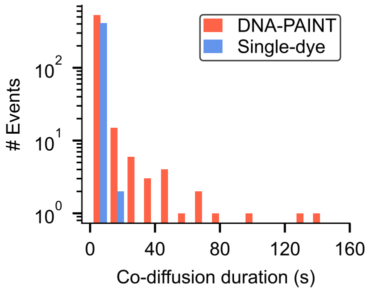

Supplementary Figure 17. FKBP dimer trajectory durations on live cells.
Histogram of detected co-diffusion durations of FKBP dimers on live cells, using Cy3B- and ATTO643 DNA-PAINT-SPT (red, $n = 17$ cells) or BG-JF5449i and BG-AF647 single-dye labeling (blue, $n = 15$ cells) with 10 nM dimerizing agent AP20187. Data collected during three-minute measurements on two identically prepared samples per labeling condition. Source data are provided.

Supplementary References

1. Patrick Binder, Nikolas D. Schnellbächer, Thomas Höfer, Nils B. Becker, and Ulrich S. Schwarz. Optimal ligand discrimination by asymmetric dimerization and turnover of interferon receptors. *Proceedings of the National Academy of Sciences*, 118(37): e2103939118, September 2021.
2. Thorben Cordes, Jan Vogelsang, and Philip Tinnefeld. On the Mechanism of Trolox as Antiblinking and Antibleaching Reagent. *Journal of the American Chemical Society*, 131(14):5018–5019, April 2009.
