# Supplementary material for: Dual-color DNA-PAINT single-particle tracking enables extended studies of membrane protein interactions
Source: Nat Commun. 2023 Jul 19;14:4345. doi: 10.1038/s41467-023-40065-8 (PMC10356854; doi:10.1038/s41467-023-40065-8)
Supplement: Supplementary file 3 — Reporting Summary [file 41467_2023_40065_MOESM3_ESM.pdf]

nature portfolio

Corresponding author(s): Kristina A. Ganzinger

Last updated by author(s): 15/06/23

Reporting Summary

Nature Portfolio wishes to improve the reproducibility of the work that we publish. This form provides structure for consistency and transparency in reporting. For further information on Nature Portfolio policies, see our Editorial Policies and the Editorial Policy Checklist.

Statistics

For all statistical analyses, confirm that the following items are present in the figure legend, table legend, main text, or Methods section.

n/a	Confirmed	
☐	☒	The exact sample size (*n*) for each experimental group/condition, given as a discrete number and unit of measurement
☐	☒	A statement on whether measurements were taken from distinct samples or whether the same sample was measured repeatedly
☒	☐	The statistical test(s) used AND whether they are one- or two-sided<br>*Only common tests should be described solely by name; describe more complex techniques in the Methods section.*
☒	☐	A description of all covariates tested
☒	☐	A description of any assumptions or corrections, such as tests of normality and adjustment for multiple comparisons
☐	☒	A full description of the statistical parameters including central tendency (e.g. means) or other basic estimates (e.g. regression coefficient) AND variation (e.g. standard deviation) or associated estimates of uncertainty (e.g. confidence intervals)
☒	☐	For null hypothesis testing, the test statistic (e.g. *F*, *t*, *r*) with confidence intervals, effect sizes, degrees of freedom and *P* value noted<br>*Give P values as exact values whenever suitable.*
☒	☐	For Bayesian analysis, information on the choice of priors and Markov chain Monte Carlo settings
☒	☐	For hierarchical and complex designs, identification of the appropriate level for tests and full reporting of outcomes
☒	☐	Estimates of effect sizes (e.g. Cohen's *d*, Pearson's *r*), indicating how they were calculated

Our web collection on statistics for biologists contains articles on many of the points above.

Software and code

Policy information about availability of computer code

Data collection	Custom written C++ software for microscopy setup control: K2 v1.0 (available upon reasonable request)
Data analysis	Custom written python code integrating trackpy v0.5.0 and Swift 0.4.3 for tracking analysis and custom written python code for co-localization analysis is available at the following repository: https://github.com/GanzingerLab/SPIT (DOI: 10.5281/zenodo.8043562).<br>FIJI 2.9.0 was used for creating ROIs. naclib 1.1.0 was used for global correction of optical distortions.

For manuscripts utilizing custom algorithms or software that are central to the research but not yet described in published literature, software must be made available to editors and reviewers. We strongly encourage code deposition in a community repository (e.g. GitHub). See the Nature Portfolio guidelines for submitting code & software for further information.

Data

Policy information about availability of data

All manuscripts must include a data availability statement. This statement should provide the following information, where applicable:

- Accession codes, unique identifiers, or web links for publicly available datasets
- A description of any restrictions on data availability
- For clinical datasets or third party data, please ensure that the statement adheres to our policy

Source data and Python notebooks to reproduce all graphs are available at Zenodo (https://doi.org/10.5281/zenodo.7290071). Due to the large file size, the raw data that support the findings of this study are available upon reasonable request. For protein structures (SNAPtag, FKBP, Antibody), PDB was used (Accession codes

3KZY, 1BL4, 1IGT (https://www.wwpdb.org/)

Human research participants

Policy information about studies involving human research participants and Sex and Gender in Research.

Reporting on sex and gender	N/A
Population characteristics	N/A
Recruitment	N/A
Ethics oversight	N/A

Note that full information on the approval of the study protocol must also be provided in the manuscript.

Field-specific reporting

Please select the one below that is the best fit for your research. If you are not sure, read the appropriate sections before making your selection.

☒ Life sciences ☐ Behavioural & social sciences ☐ Ecological, evolutionary & environmental sciences

For a reference copy of the document with all sections, see nature.com/documents/nr-reporting-summary-flat.pdf

Life sciences study design

All studies must disclose on these points even when the disclosure is negative.

Sample size	Sample size was not determined beforehand but decided based on technical feasibility (data storage, time and resources) and sample sizes used in similar studies. Conclusions were based on data sampled from several thousand individually localized and tracked single molecules: For in-vitro experiments, we performed automatized single-molecule localization and frame-by-frame tracking to build up trajectories from 32 supported lipid bilayer specimen, thereby collecting individual data from thousands of molecules. For live cell imaging, we detected the outline of 259 cells, performed automatized localization and frame-by-frame tracking to build up trajectories of thousands of molecules on the surface of the cells.
Data exclusions	Trajectories shorter than 10 frames (too few data points for meaningful statistical analysis), or with diffusion constants smaller than 0.01 um2/s (immobile molecules) were rejected. Co-localization events shorter than 10 frames were rejected on the basis that random Brownian motion will also result in co-localization on these time scales.
Replication	Replicates were used for error estimation. For both live cell and in vitro experiments, extra replicates were prepared in case bilayer preparation failed. This was the case in about 10% of the samples. Exact number of replicates are stated in figure legends (typically experiments were performed 5 times with similar results).
Randomization	The nature of the experiment (in-vitro single-molecule study) eliminates the need for randomization.
Blinding	No blinding was performed as data was fully analysed by an automated data analysis pipeline with identical parameter settings for datasets that were subsequently compared.

Reporting for specific materials, systems and methods

We require information from authors about some types of materials, experimental systems and methods used in many studies. Here, indicate whether each material, system or method listed is relevant to your study. If you are not sure if a list item applies to your research, read the appropriate section before selecting a response.

Materials & experimental systems

n/a	Involved in the study	
☐	☒	Antibodies
☐	☒	Eukaryotic cell lines
☒	☐	Palaeontology and archaeology
☒	☐	Animals and other organisms
☒	☐	Clinical data
☒	☐	Dual use research of concern

Methods

n/a	Involved in the study	
☒	☐	ChIP-seq
☒	☐	Flow cytometry
☒	☐	MRI-based neuroimaging

Antibodies

Antibodies used	ChromoTek SNAP/CLIP-tag® Monoclonal antibody (cat no: 6F9, clone no: 6F9), Chromotek.<br>FluoTag-Q anti-GFP single-domain antibody (cat no: N0305, clone no: 1H1, Nanotag Biotechnologies)
Validation	ChromoTek: Western blot analysis of cell extract from HEK293T cells transiently expressing SNAP-Tubulin and from untransfected cells. (https://www.citeab.com/antibodies/7544966-6f9-snap-clip-tag-antibody-6f9?des=5e766bd554da5f99)<br>Nanotag: No further validation is given by the manufacturer (https://www.citeab.com/antibodies/10334608-n0301-fluotag-q-anti-gfp)

Eukaryotic cell lines

Policy information about cell lines and Sex and Gender in Research

Cell line source(s)	Jurkat E6-1 (ATCC TIB-152), Cell lot number 70007821, male<br>HEK293T (ATCC CRL-3216)
Authentication	Cells used were acquired directly from ATCC
Mycoplasma contamination	tested negative
Commonly misidentified lines (See ICLAC register)	No commonly misidentified cell lines were used.
