# Supplementary material for: Dual-color DNA-PAINT single-particle tracking enables extended studies of membrane protein interactions
Source: Nat Commun. 2023 Jul 19;14:4345. doi: 10.1038/s41467-023-40065-8 (PMC10356854; doi:10.1038/s41467-023-40065-8)
Supplement: Supplementary file 4 — Description of Additional Supplementary Files [file 41467_2023_40065_MOESM4_ESM.pdf]

Description of Additional Supplementary Files

Supplementary Movie 1. SLB with reconstituted FKBP proteins labeled with dual-color DNA-PAINT. TIRFM video (40 ms exposure time, replayed at 25 fps) of reconstituted FKBP proteins in monomer-dimer equilibrium labeled with dual-color DNA-PAINT. Colocalization events are marked with a circle. Scale bar 10 µm.

Supplementary Movie 2. Single-molecule tracking and detection of co-diffusing trajectories. Trajectories of molecules displayed in Supplementary Movie 1 with co-diffusing trajectories shaded in yellow. Scale bar 10 µm.

Supplementary Movie 3. Association and dissociation of a dimer. TIRFM video (40 ms exposure time, replayed at 25 fps) of reconstituted dimerized FKBP proteins labeled with dual-color DNA-PAINT. Dimerization is induced by anti-SNAPtag antibodies. Two FKBP monomers associate (white circles, association at ≈ 2.4 s), co-diffuse (blue circle and track) for about 21 s, and dissociate into two monomers (white circles, dissociation at ≈ 23.5 s. Scale bar: 4 µm.

Supplementary Movie 4. Screening of passivation methods. TIRFM video (80 ms exposure time, replayed at 25 fps) of dual-color DNA-PAINT-SPT of membrane proteins and GFP signal of adhered cells on different surfaces. Field of view: 20 µm.

Supplementary Movie 5. DNA-PAINT labeled membrane proteins diffusing on Jurkat T cell. TIRFM video (80 ms exposure time, replayed at 25 fps) showing single-molecule trajectories of DNA-PAINT labeled membrane proteins expressed on a Jurkat T cell. Localized molecules (yellow boxes) and trajectory of an individual molecule (red box and trajectory). Scale bar: 5 µm.

Supplementary Movie 6. Dual-color DNA-PAINT labeled FKBP proteins on Jurkat T cell. TIRFM video (100 ms exposure time, replayed at 25 fps) of FKBP proteins diffusing on a Jurkat T cell membrane (green and magenta circles), labeled with dual-color ATTO643- and Cy3B-DNA-PAINT-SPT. A FKBP dimer observable for > 1 minute is highlighted (blue trajectory). Scale bar: 5 µm.

Supplementary Movie 7. Dual-color DNA-PAINT labeled membrane proteins diffusing on Jurkat T cell. TIRFM video (80 ms exposure time, replayed at 25 fps) of membrane proteins expressed on a Jurkat T cell and labeled orthogonally with dual-color DNA-PAINT-SPT. Scale bar: 10 µm.
